# Automation of flow injection gas diffusion-ion chromatography for the nanomolar determination of methylamines and ammonia in seawater and atmospheric samples

**DOI:** 10.1155/S1463924695000320

**Published:** 1995

**Authors:** Stuart W. Gibb, John W. Wood, R. Fauzi, C. Mantoura

**Affiliations:** 1 Plymouth Marine Laboratory Prospect Place West Hoe Plymouth PL1 3DH UK; 2 University of East Anglia University Plain Norwich NR4 7TJ UK; 3 Marine Biological Association Citadel Hill Plymouth UK

## Abstract

The automation and improved design and performance of Flow Injection Gas Diffusion-Ion Chromatography (FIGD-IC), a novel technique for the simultaneous analysis of trace ammonia (NH_3_) and methylamines (MAs) in aqueous media, is presented. Automated Flow Injection Gas Diffusion (FIGD) promotes the selective transmembrane diffusion of MAs and NH_3_ from aqueous sample under strongly alkaline (pH > 12, NaOH), chelated (EDTA) conditions into a recycled acidic acceptor stream. The acceptor is then injected onto an ion chromatograph where NH_3_ and the MAs are fully resolved as their cations and detected conductimetrically. A versatile PC interfaced control unit and data capture unit (DCU) are employed in series to direct the selonoid valve switching sequence, IC operation and collection of data. Automation, together with other modifications improved both linearily (R^2^ > 0.99 MAs 0-100 nM, NH_3_ 0-1000 nM) and precision (<8%) of FIGD-IC at nanomolar concentrations, compared with the manual procedure. The system was successfully applied to the determination of MAs and NH_3_ in seawater and in trapped particulate and gaseous atmospheric samples during an oceanographic research cruise.


Journal of Automatic Chemistry, Vol. 17, No. 6 (November–December 1995), pp. 205–212

### Automation of flow injection gas diffusion–ion chromatography for the nanomolar determination of methylamines and ammonia in seawater and atmospheric samples

**Stuart W. Gibb[1,2], John W. Wood[3], R. Fauzi[1] and C. Mantoura[1]**

[1] *Plymouth Marine Laboratory, Prospect Place, West Hoe, Plymouth PL1 3DH, UK.* [2] *University of East Anglia, University Plain, Norwich NR4 7TJ, UK.* [3] *Marine Biological Association, Citadel Hill, Plymouth, UK*

*The automation and improved design and performance of Flow Injection Gas Diffusion-Ion Chromatography (FIGD-IC), a novel technique for the simultaneous analysis of trace ammonia ($NH_3$) and methylamines (MAs) in aqueous media, is presented. Automated Flow Injection Gas Diffusion (FIGD) promotes the selective transmembrane diffusion of MAs and $NH_3$ from aqueous sample under strongly alkaline (pH > 12, NaOH), chelated (EDTA) conditions into a recycled acidic acceptor stream. The acceptor is then injected onto an ion chromatograph where $NH_3$ and the MAs are fully resolved as their cations and detected conductimetrically. A versatile PC interfaced control unit and data capture unit (DCU) are employed in series to direct the selonoid valve switching sequence, IC operation and collection of data. Automation, together with other modifications improved both linearity ($R^2 > 0.99$ MAs 0–100 nM, $NH_3$ 0–1000 nM) and precision (<8%) of FIGD-IC at nanomolar concentrations, compared with the manual procedure. The system was successfully applied to the determination of MAs and $NH_3$ in seawater and in trapped particulate and gaseous atmospheric samples during an oceanographic research cruise.*

### Introduction

Nitrogen, a bio-essential element in the marine environment [1, 2], is found in a variety of inorganic and organic forms in oxic seawater, ranging from the thermodynamically most stable species, nitrate ($NO_3^-$), to the reduced compounds such as ammonia ($NH_3$) and its mono-, di-, and tri-methylamine derivatives ($CH_3NH_2$, $(CH_3)_2NH$ and $(CH_3)_3N$, abbreviated MMA, DMA and TMA respectively). Methylamines (MAs), like $NH_3$ are polar, volatile, water soluble species which undergo extensive hydrogen bonding to form basic solutions ($pK_a = 9.25$–$10.77$). Due to their low molecular weight, ability to participate in phase transfer processes [3–6] and importance in marine nitrogen fertility [1, 7], detoxification and osmoregulation [8–10], $NH_3$ and MAs are widely distributed and dynamic within the marine environment.

Due to their volatility, $NH_3$ and the MAs (boiling points −33·4–7·4°C) are also capable of gaseous evasion across the air–sea interface, thus introducing alkali and reduced nitrogen into the troposphere [3, 4, 11]. Here, through processes such as their dissolution into cloud or rainwater, reaction with $H_2SO_4$ aerosol particles and photochemical oxidation to $NO_x$, $NH_3$ and the MAs may influence the redistribution of both nitrogen and sulphur, the acid-base chemistry of the atmosphere and, ultimately, climatic parameters such as the number density and chemical composition of cloud condensation nuclei [3, 4, 11–13].

Methylamines are of additional importance due to the conversion of secondary amines (for example DMA) to their *N*-chloro-derivatives in chlorine disinfected wastewaters [14] and the implication of secondary and tertiary amines in the synthesis of carcinogenic nitrosamines in aqueous media, air, soils and foodstuffs [15].

Aspects of the marine biogeochemistry of $NH_3$ and MAs have been studied over many years (for example [3, 5, 8]). However, understanding their distribution and transformations has been restricted by the absence of an analytical technique capable of their selective quantification at the nanomolar levels typical of the marine environment.

Ammonia is often determined in natural waters potentiometrically by ion-selective electrodes [16, 17], or colorimetrically, typically by a version of the indophenol blue method [18]. However, away from bacterially active, anoxic or anthropogenically influenced regions, concentrations of $NH_3$ often fall below the sensitivity of these techniques (~0·1 μM). Only recently have cathodic stripping voltammetry [19] and *o*-phthalaldehyde (OPA) [20] fluorescence techniques with limits of detection < 10 nM been reliably applied to the analysis of ammonia in pristine and open oceanic waters.

Methylamines, meanwhile, are normally studied by Gas or High Performance Liquid Chromatography (GC or HPLC). In practice, many GC techniques suffer from peak asymmetry [21–23], ghosting phenomena [24–26] and detector response quenching [21, 23, 27], while HPLC techniques require derivatization of MAs before detection. While derivatization is advantageous in overcoming the often problematic polar, volatile nature of the MAs, there is no single derivatizing agent available for primary, second and tertiary amines. Only in ion chromatography (IC) are these problems overcome and it is possible to simultaneously analyse ammonia and MAs (as solvated ammonium ($NH_4^+$) and methylammonium cations) [21, 28, 29].

The authors recently described a novel technique, Flow

0142-0453/95 $10.00 © 1995 Taylor & Francis Ltd.

Injection Gas Diffusion coupled to Ion Chromatography (FIDG-IC), which exploits the advantages of IC and permits the simultaneous analysis of $NH_3$ and the MAs at nanomolar levels by a single analytical technique in natural waters [30]. This paper reports on the improvement, automation and computer interfacing of the technique which has increased its precision and reliability. The applicability of the technique is demonstrated through the analysis of MAs and ammonia in seawater and atmospheric samples during a research cruise in the northwestern Indian Ocean.

### System design

### *Reagents*

Ammonia and MA stock solutions (0·10 M) were prepared from their hydrochloride salts (Fluka). Single and mixed standards were prepared daily from these stocks. Internal standards (ISs) cyclo-propylamine (c-PA) and sec-butylamine (s-BA) were prepared from serial dilution of laboratory grade reagents (Sigma, UK). Cyclo-PA was chosen as an IS since its occurrence in the natural environment has not been reported.

The alkaline-chelating reagent, 1·1 M ethylene diamine *N,N,N′,N′*, tetra-acetic acid (EDTA)/0·11 M NaOH was prepared from the tetra-sodium salt of EDTA and ACS grade sodium hydroxide pellets (Sigma, UK). Eluent (40 mM MSA) was routinely prepared via a 1·0 M stock from 'Aristar' grade concentrate (BDH, UK).

Water taken freshly from a Milli-Q Water Purification System (Millipore, UK) with a specific resistivity of $>18\ M\Omega$ and further passed through a sealed ion-exchange column packed with Amberlite IR-120+ (proton form) was used as the diluent or solvent in the preparation of standards and reagents. Further ion-exchange was used to reduce the interference of alkali metals ($Na^+$ and $K^+$) and the contribution of blanks ($NH_3$ and MAs).

### *Ion chromatograph and suppression system*

Chromatography was performed on a Dionex DX-100 IC equipped with conductimetric detection (Dionex, UK). Resolution of MAs was effected under isocratic elution (40 mM Methane Sulphonic Acid, MSA, 1 ml/min) by two IonPac CG-10 cation-exchange columns (surface sulphonated, cross-linked styrene–divinyl benzene; 4 × 50 mm; Dionex, UK). A Dionex Cation Self Regenerating Suppressor (CSRS) operated with Self-Regenerating Suppressor (SRS) current controller (Dionex, UK) was used to chemically suppress the background conductivity. Injections were carried out by a pneumatically actuated injection valve fitted with a 200 μl injection loop.

### *Flow injection gas diffusion system*

The diffusion and stripper cells were custom designed and fabricated from milled perspex [30]. Each composite module consists of a pair of mirror image blocks into which a zig-zag shallow rectangular cross-section channel was cut (length 1004 mm, width 2·0 mm, depth ~0·1 mm). The two blocks were secured using stainless-steel screws and reproducibly and uniformly tightened using a calibrated torque-limiting screwdriver.

Microporous PTFE was used as a the gas-exchange membrane (Goretex MF/002/PM–pore size 1 μM, thickness 0·076 mm, porosity 78%; W. L. Gore, UK). Supplied in sheet form, this material was cut to a template while sandwiched between sheets of paper to facilitate easy handling.

A full analytical description and evaluation of the original FIGD-IC technique has previously been published [30].

A series of normally closed solenoid operated valves were used to control and direct liquid flow in the FIGD system (Biochem Valve Corp., USA).

(1) One four-inlet/one-outlet isolation mixing valve (080T4 12-62-4 pps, 0·062 in inlet) used to switch between sample, standard and wash solutions as required (see figure 1, VI).
(2) Two two-inlet/one-outlet isolation mixing valves (08T2 12-54-4 pps, 0·054 in inlet) coupled together to effect switching of the acceptor stream from enrichment to transfer (see figure 1, V2/V3).
(3) One three-way normally closed isolation valve (075T3MP 12-32-3 PEEK, 0·032 in inlet (see figure 1, V4).

Two single-speed, dual-channel peristaltic pumps (Ismatec type CA-2E 840, Ismatec, Switzerland) fitted with compatible pump tubing were used to generate liquid flow. All other flow lines were of Teflon (0.3, 0.5 and 0.7 mm i.d.) connected with Teflon faced grippers and 1/26 screw Tefzel end fittings and couplers (Anachem, UK). Diffusion times employed in the analysis of standards and samples were 20–60 min.

### *Software, PC and automation*

The analogue output signal (10 μS range; 10% offset/1 V output) from the IC was converted to digital (2 Hz) using a Philips PU6031/10 data collection unit (DCU) and then processed using ATI-Unicam 4880 chromatographic software run on a Compaq 4/25 portable PC within a Windows environment. The 4880 software was also used to predetermine the timed events of valve and IC switching.

*Control of solenoid valves:* The DCU has seven external reed relays which are individually addressable from within the 4880 software, each with a set of uncommitted normally open contacts and available for external operation. The contacts are light duty and unsuitable for direct operation of the solenoid valves. It is thus necessary either to provide additional heavier duty relays, or more efficiently, to use an electronic switching circuit. The latter approach is advantageous since the circuit can be designed to be more flexible and made able to handle open collector (transistor switched) or TTL levels, as well as contact closure inputs. The solenoid valves are not then limited to use with the DCU, but could alternatively be operated by digital control systems.

The electronic interface is quite simple and is shown in

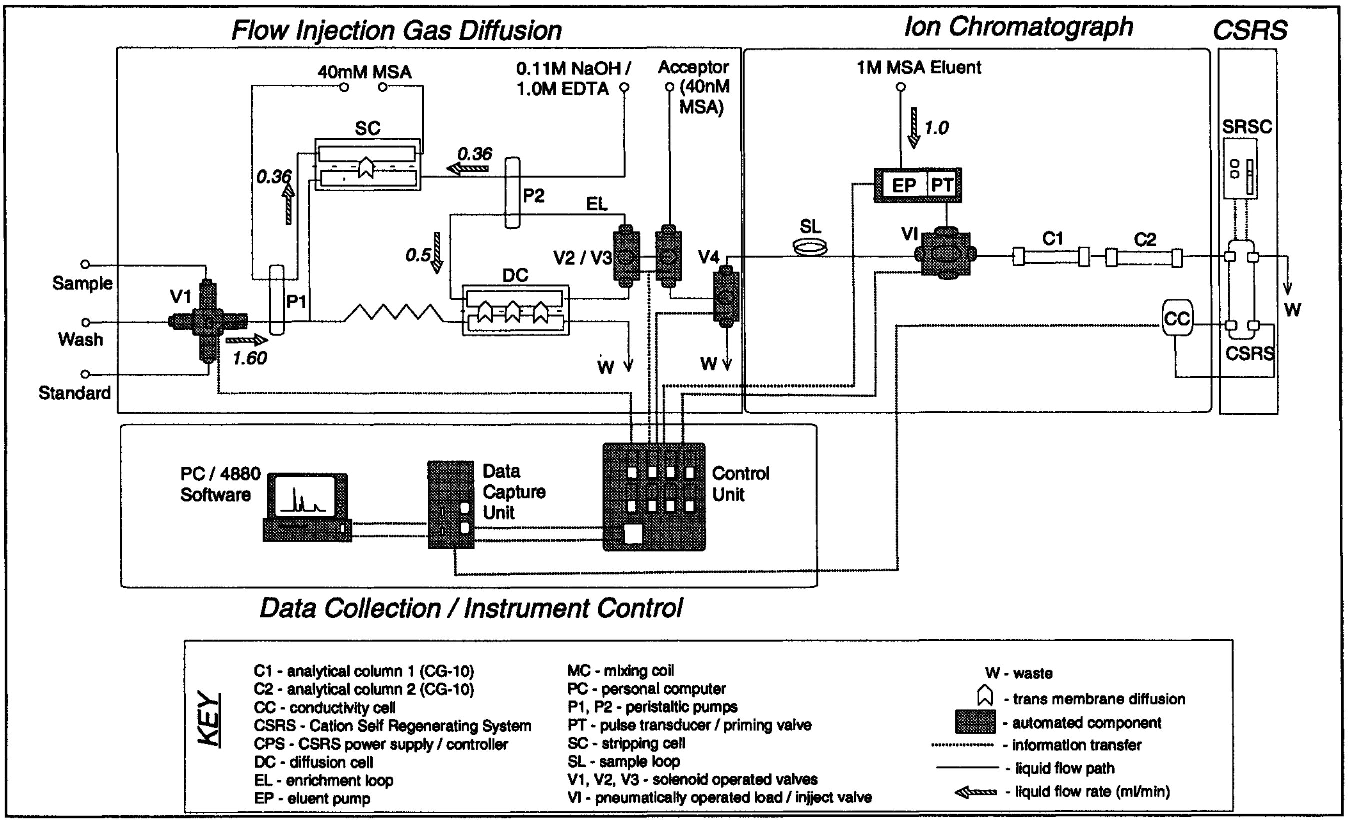

*Figure 1. Schematic of the automated Flow Injection Gas Diffusion-Ion Chromatography System (FIGD-IC).*

figure 2 in a form suitable for individual control of the 4 solenoids of valve V1 (see figure 4), and for the single solenoid of valve V3 (see figure 1).

The control input is connected to a Schmitt NAND gate (four of which are available in one CMOS 4093 series package). The output of the gate is connected to a high current VMOS field effect transistor which acts as the switch for turning the solenoid on and off. When the input reed relay is open, the two inputs of the NAND gate are at logical 1 due to the two resistors which act as 'pull-ups' to the 5 V line. The output of the NAND gate will therefore be at logical 0, the transistor will be turned off, and the solenoid will not be energized. The diode in parallel with the solenoid prevents the build-up of back e.m.f. when the solenoid is turned off.

When the reed relay contacts close, after a software command, one input of the NAND gate will go to logical 0 thus forcing the output to go to logical 1. This turns on the transistor and the solenoid will be energized. The action is the same if the 'contact closure' is performed by an open collector transistor, or the control line is forced to logical 0 by a TTL logic level. The interface is therefore quite versatile. The use of a Schmitt NAND gate ensures positive on–off switching, even if the control input changes slowly. The built-in hysteresis of this gate prevents solenoid 'chatter' during switching, which would generate considerable electrical and mechanical noise.

The development of the circuit for use with values V2 and V3 is shown in figure 3, where pairs of solenoids are operated simultaneously in a combination of two, two-port valves. These are interconnected to control the enrich or flush/discharge cycle, and enable the flow to be switched between cycles by a single timed event in the software.

When the control contacts are open, solenoids S1 and S2 are released but, due to the inverting action of a second VMOS field effect transistor, solenoids S3 and S4 are energized. It follows that when the control contacts close, S1 and S2 are energized and S3 and S4 are released.

*Remote IC injection:* To facilitate the automatic injection of the sample, another simple interface is required. This interface must allow the control signal to operate a pneumatic valve on the IC which is triggered by linking two electrical contacts. While this could be performed by the control contacts directly, it was felt useful to preserve the 'universal' nature of the interface and arrange for the link to be made by a VMOS field effect transistor. This could then be controlled by an open collector or TTL logic level as before. The circuit of a suitable interface is shown in figure 3 (note that this is very similar to figure 2).

*Practical considerations:* Power to operate the solenoid valves is provided by a 12 V, 2·5 A regulated power supply. The supply and the solenoid driver circuits (built on custom-printed circuit boards) are all housed in a single metal enclosure. Connections to the DCU and solenoid valves are routed through multicore cable and 25-way D connectors. Switches are provided so that all of the valves can be operated manually if required, and current-limited light-emitting diodes (LEDs) are connected in parallel with each solenoid to indicate status (illuminated when the solenoid is energized).

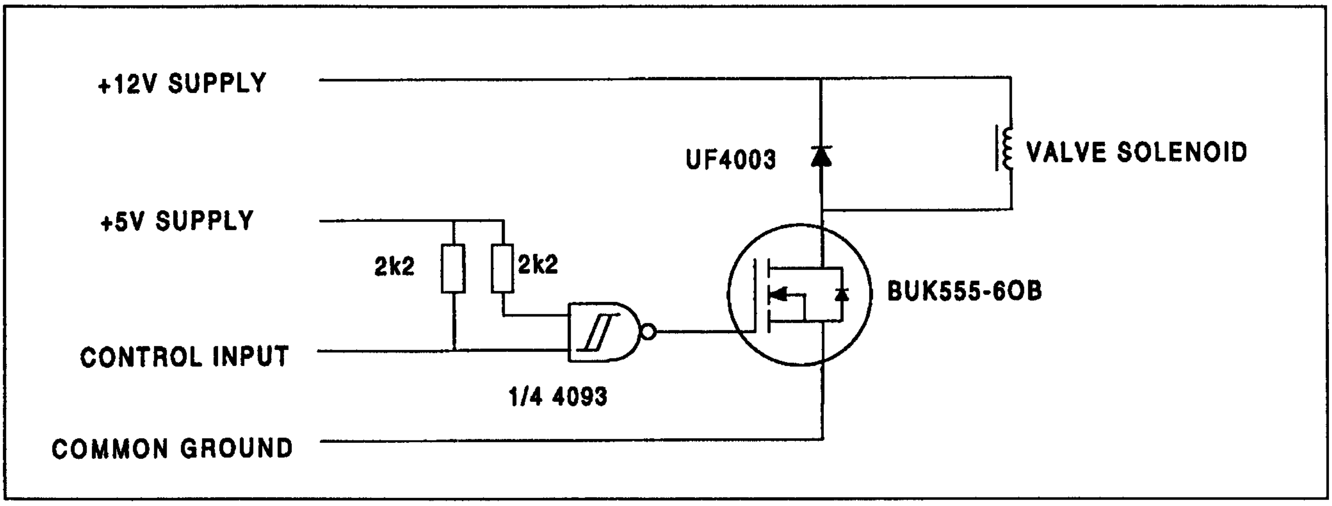

*Figure 2. One channel of solenoid driver for independent solenoid control; four channels needed for valve V1 (0804T) and one for V4 (0705T3).*

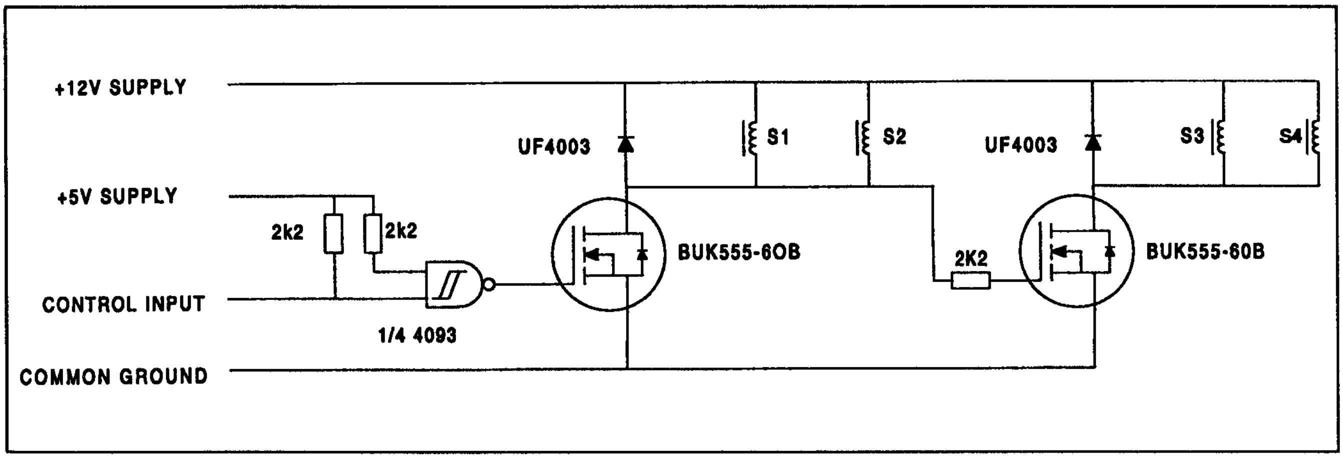

*Figure 3. Addition of inverter for control of coupled valves V2 and V3 (2 × 080T2).*

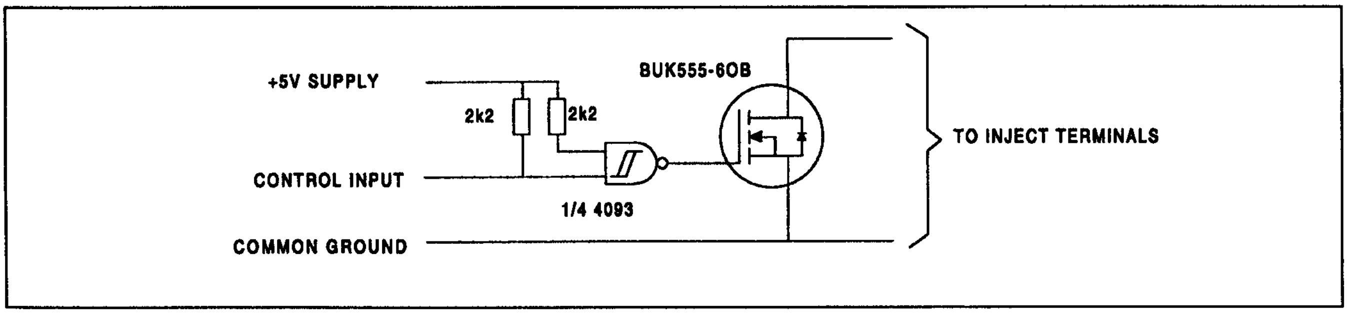

*Figure 4. Contact closure simulator for remote inject of IC.*

### Outline of the technique

In the automated FIGD-IC system (see figure 1), seawater is pumped continuously and treated with the mixed EDTA (1·0 M)/NaOH (0·11 M) reagent to chelate alkaline earth cations ($Ca^{2+}$, $Mg^{2+}$, $Be^{2+}$, and $Ba^{2+}$) and, at the same time, to raise the pH of the mixture to $> 12$. Under these conditions, $> 95\%$ of the total dissolved $NH_4^+$ and MAs are deprotonated to their volatile gaseous forms and capable of diffusion from the sample stream, across the hydrophobic diffusion cell membrane, into an acidic acceptor stream (40 mM MSA) in which they are re-protonated. Recycling the acceptor, *via* coupled valves V2 and V3, promotes selective accumulation of the analytes in the enrichment loop. Following an enrichment period of between 20 and 60 minutes, the amine enriched accept is transferred to the IC via valve V4 and 200 µl is injected. Analytes and internal standards are chromato-

*Table 1. Regression data for automated FIGD-IC (spiked seawater) and comparison of response linearity and FSDs for manual and automated FIGD-IC systems.*

| | Parameter | Species | | | | |
|---|---|---|---|---|---|---|
| | | $NH_3$ | MMA | DMA | c-PA | TMA |
| Regression data | Regression range (nM) | 0–1000* | 0–100 | 0–100 | 0–100 | 0–100 |
| | No. of points, $N$ | 5 | 5 | 5 | 5 | 5 |
| | Normalized response† | 3·12 | 2·87 | 2·48 | 1·00 | 2·09 |
| | Normalized intercept† | 61·8 | 2·32 | 1·75 | 1·00 | 2·33 |
| Linearity ($R^2$) | Manual system (0–1000 nM)** | 0·988 | 0·992 | 0·992 | 0·996 | 0·990 |
| | Automated system | 0·992 | 0·997 | 0·992 | 0·998 | 0·992 |
| Relative Standard Deviation (% at 100 nM spike) | Manual system** | 10·5 | 6·09 | 4·06 | n<a | 5·09 |
| | Automated system | 7·99 | 6·86 | 2·77 | 3·83 | 2·14 |
| Limit of detection (nM) | Manual system (20 min diffusion) | 20–40 | ~5 | 3–4 | n/a | 3–4 |
| Automated system (40 min diffusion) | | 30 | 4 | 1–2 | n/a | 1–2 |

All data Gulf of Oman except * Ammonia regression data, which were English Channel seawater [21]; ** Spiked Mediterranean deep water ( = 3); † normalized wrt c-PA (IS); n/a not applicable.

graphically resolved using isocratic elution (40 mM MSA). The monovalent cations are conductimetrically detected with chemical suppression of the background signal. The analogue signal (which is proportional to the analyte concentration) is digitized by the DCU and collected and processed by the 4880 software system.

Since FIGD-IC response has previously been shown to relate linearly to diffusion time ($R^2 > 0{\cdot}99$, 1–60 min [21]), diffusion times can be selected in accordance with analytes concentration. As a consequence, with the appropriate switching sequence, utilization of diffusion or enrichment times in excess of the 15-min chromatographic run time, permits enrichment of one sample and chromatography of the preceding sample to proceed in parallel [5]. This significantly increases sample throughput and operational efficiency.

Chemical suppression systems, such as the CSRS employed here, greatly enhance signal to noise ratio by effectively decreasing background eluent conductivity, increasing relative analyte detector response and eliminating system peaks by removing counter ions. The result is a significant improvement in analyte detection limits. CSRS is a high capacity, automatic suppression system in which the use of detector cell effluent as a water source eliminates the need for chemical regenerant solutions or ion-exchange cartridges. With a dead volume of only 30 µl analyte dispersion effects are minimal.

Since all reagent and sample bottle headspace is fed with acid-scrubbed air fed through PTFE lines, atmospheric contamination is minimized and reagent life is prolonged. Using a second diffusion or 'stripper' cell (SC in figure 1) minimizes the $NH_3$ and MAs' content of the EDTA/NaOH reagent before its addition to the sample. Eluent and FIGD acceptor solution (both 40 mM MSA) are prepared concurrently, ensuring the two are matched and therefore minimizing IC base-line perturbation upon acceptor injection.

The two internal standards incorporated into the analytical scheme were used to monitor FIGD-IC performance (figure 1). *Sec*-BA, added to the acceptor (typically 10 µM), to monitor IC stability and reproducibility, while c-PA spiked into the sample was generally used to quantify determinant concentrations through predetermined relative response factors. It was also possible to determine a range of other low molecular weight alkylamines from aqueous samples [21].

### Calibration, precision and sensitivity

The automated FIGD-IC system gave a highly linear response to $NH_3$ and MAs in both standards and spiked seawater with relatively good precision in the concentration range of interest (see table 1). Both $R^2$ and RSD coefficients show, in general, an improvement over those achieved with the manual system. This is particularly important in the trace determination of MAs.

On-column detection limits for IC alone were 0·20 ng $NH_4^+$, 0·37 ng MMA, 0·54 ng DMA, 0·95 nf TMA and 0·72 ng c-PA for a 200 µl injection volume [21]. Limits of detection for the coupled FIGD-IC system are dependent upon the diffusion time employed, base-line stability, the relative abundance of analytes and the influence of the system blank. Since $NH_3$, in particular, gives a significantly non-zero blank, this makes trace analysis of $NH_3$ more difficult than for the MAs. In practise, improvements in MA detection limits (but not $NH_3$) are achieved using the automated system with a 40 min diffusion time (table 1). Detection limits are improved most noticeably for DMA and TMA, since these occur in regions of greater base-line stability.

### Applications

The automated FIGD-IC system was deployed for the analysis of $NH_3$ and MAs during Cruises 210 and 212 of

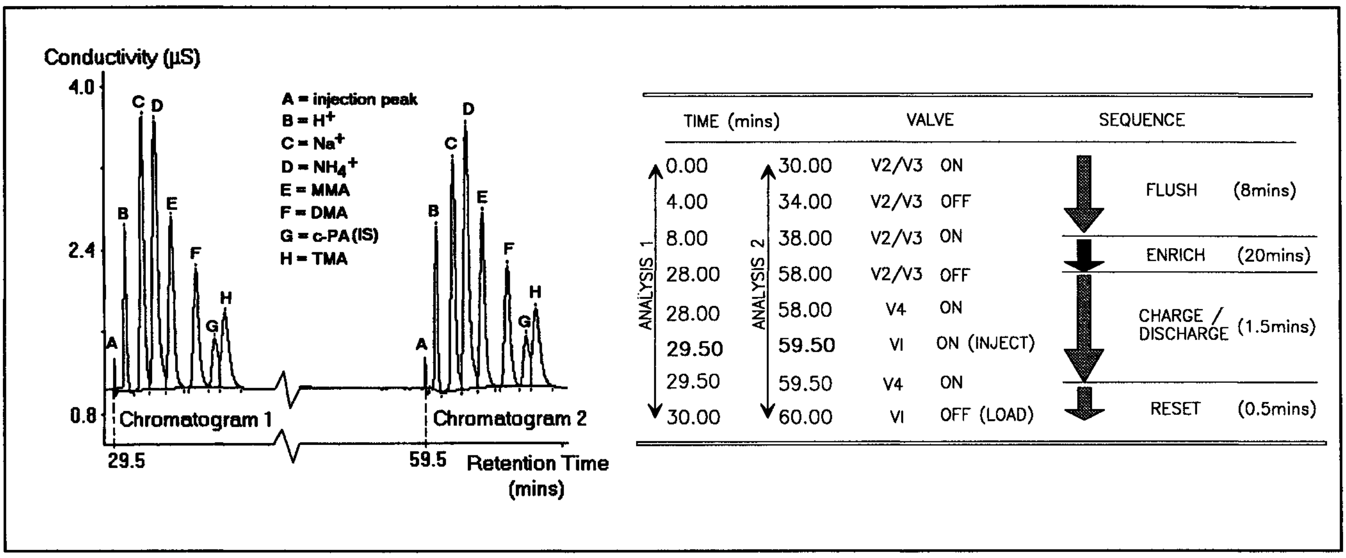

*Figure 5. Automared FIGD-IC chromatogram and corresponding valve switching sequence for duplicate analysis of a seawater sample spiked 1 µM in $NH_3$, MAs and c-PA (IS) based on a 20 min diffusion.*

RRS *Discovery* in support of the UK's ARABESQUE programme as part of the Joint Global Oceanic Flux Study (JGOFS) community research programme in the Arabian Sea (August–December 1994).

### *Seawater samples*

Seawater samples were collected in gas tight bottles, either directly from a CTD rosette (equipped with 12 bottle sampler, chlorophyll fluorimeter, oxygen electrode, transmissometer and underwater light meter) or from an on-line submersible pump (at 2 m depth). Samples were spiked with c-PA (typically 20 nM) and analysed unfiltered using 20–60 min diffusion times.

### *Atmospheric samples*

Particulate and gaseous phase atmospheric samples were collected in tandem using a cyclonic filter air-sampling technique [31] adapted for use with automated FIGD-IC. Aerosols were collected on PTFE pre-filters (47 mm diameter, 1 µM poresize; Costar, UK), while gaseous species were collected downstream on pairs of oxalic acid-soaked paper filters (47 mm diameter, Whatman 40, Whatman Scientific, UK). Samples were collected in triplicate over periods of 10–100 h (at ~50 $dm^3$/min) from a height of ~8 m above sea level. The exact volume of air sampled was determined by in-line gas meters (B.S.S., UK). Filters were extracted [31] and diluted in MQ water (to 50 ml) and analysed by FIGD-IC using a 20 min diffusion time. Both particulate and gaseous concentrations were background corrected using 'blank' filters treated in the same manner as the sampled filters but without air pumping (see figure 6).

### Results

The automated FIGD-IC system was successfully applied to the determination of $NH_3$ and MAs at nanomolar

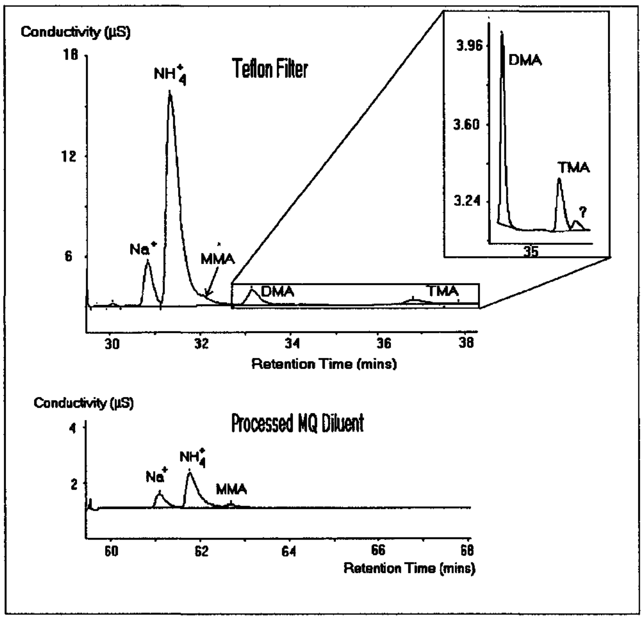

*Figure 6. FIGD-IC chromatograms for a MQ water extracted Teflon filter and for the diluent only.*

concentrations in a seawater (see figure 7) and in trapped atmospheric samples (table 2). However, in atmospheric samples, the relative abundance of $NH_3$ with respect to MMA made quantification of MMA difficult since it appeared only as a shoulder to the $NH_2$ peak (figure 6). Furthermore it was not possible to quantify DMA in the gaseous phase due to an interfering peak arising from processing the acid soaked paper filter. In general, $NH_3$ was the dominant species occurring at 5–1000 times greater concentrations than those of any MA (whose levels occasionally fall below the detection limits of the system).

Monomethylamine was generally observed to be the dominant MA in Indian Ocean seawater, while TMA was normally detected at concentrations of <5 nM. Highest concentrations in oceanic vertical profiles were

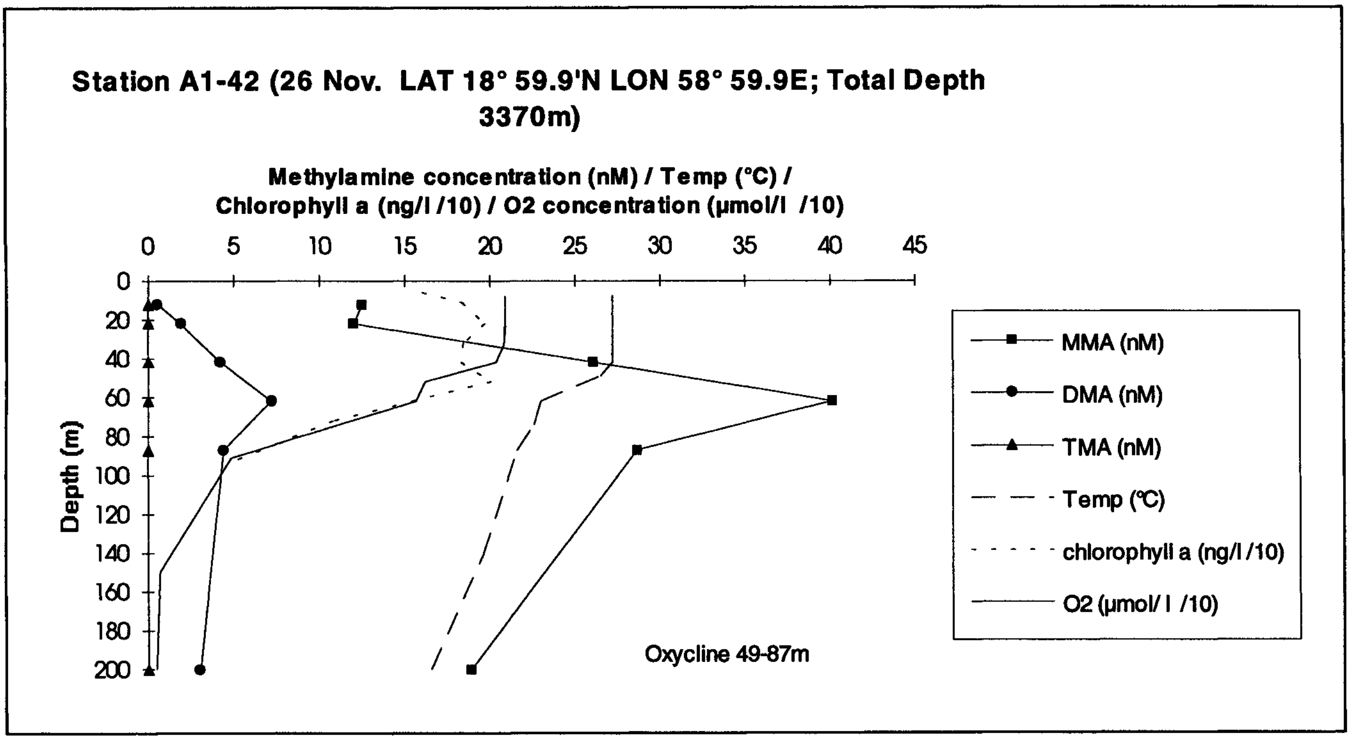

*Figure 7. Depth profile of MA concentrations at 'ARABESQUE' sampling station A1-42* (RRS Discovery *Cruise 210*).

*Table 2. Selected particulate and gaseous phase atmospheric concentrations for the northwestern Indian Ocean (August–December 1994) determined by automated FIGD-IC.*

| Phase | Concentration range ($pmol/m^3$) (No. of samples) | | | |
|---|---|---|---|---|
| | $NH_3$ | MMA | DMA | TMA |
| Particulate | 940–4880 | 49–190 | 50–228 | 0–14 |
| (1 µM Teflon filter) | (9) | (7) | (9) | (9) |
| Gaseous | 350–3190 | 36–169 | n/a | 0–9 |
| (Acid-soaked paper filter) | (9) | (8) | | (8) |

generally observed in the regions of the thermocline and greatest primary production (shown as 'Temperature' and 'Chlorophyll a' concentration respectively in figure 7).

### Comparison with other analytical techniques

Techniques for the analysis of nanomolar concentrations of MMA, DMA and TMA generally require prolonged preconcentration times (up to 36 h) [24, 32, 33], also they are labour intensive [21, 32, 33] or they are unsuitable for use on ship [32]. FIGD-IC, on the other hand, allows determination of up to four samples per hour and is also less susceptible to contamination than the other techniques.

The prerequisite of a chromatographic step for simultaneous determination of $NH_3$ and MAs, results in a considerably lower sample throughput than may be achieved for a single determinant. Recently it has been possible to determine $NH_3$ by OPA-fluorimetry using flow injection principles and a gas diffusion cell [20]. This technique gives a sensitive (~2 nM) and precise (2%) analysis, and, with a throughput of up to 60 samples/hour, is ideally suited to high resolution, 'real' time analysis of $NH_3$. The only major drawback of this technique is that primary amines (for example MMA) are positive interferents. The ability to chromatographically resolve these analytes is an advantage of FIGD-IC. (Note that previous studies have shown manual FIGD-IC to be in good agreement with OPA fluorimetry [20] for the determination of $NH_3$ [$R^2$ = 0·995, **n** = 24; slope = 0·904, intercept = 8 nM].)

### Conclusion

The automated FIGD-IC system proposed in this paper permits the near real-time determination of MAs and $NH_3$ in seawater and aqueous extracts of atmospheric gaseous and particulate samples. Simultaneous analysis of nanomolar concentrations are possible without the need for derivatization schemes or lengthy preconcentration procedures. While the manual FIGD-IC system required constant supervision (for switching valves, injecting samples etc.), automation significantly reduced this need. Automation improved the analytical reproduciblity and sample throughput of the system by increasing precision and accuracy valve switching and by reducing the scope for human error.

The flexible nature of the automation control system described allows considerable scope for broader applications of the FIGD-IC and also for other automated analytical applications, for example other natural waters such as rainwater and fresh waters.

### Acknowledgements

This work was carried out with the financial support of the Natural Environment Research Council of the UK under grant GR3/8715. The authors would like to extend their thanks to Akis Katsakoglou of W. L. Gore and Associates and to Maria Walton of Dionex (UK).

### References

1. Riley, J. P. and Chester, R., *Introduction to Marine Chemistry*. Academic Press, London (1971).

2. Carpenter, E. J., and Capone, D. G., *Nitrogen in the Marine Environment*. Academic Press, London (1983).

3. Van Neste, A., Duce, R. A., and Lee, C., *Geophysical Research Letters*, **14**, 7 (1987), 711.

4. Quinn, P. K., Charlson, R. J. and Zoller, W. H., *Tellus*, **39B** (1987), 413.

5. Lee, C., In *Nitrogen Cycling in Coastal Marine Environments* (*SCOPE 33*). Blackburn, T. H. and Sorenson, J. (Eds). John Wiley and Sons, Chichester (1988).

6. Wang, X.-U. and Lee, C., *Geochimica et Cosmochimica Acta*, **54** (1990), 275.

7. Woodwell, G. M., In *Fundamentals of Aquatic Ecosystems*. Barnes, R. S. K., and Mann, K. H. (Eds). Blackwell Scientific, Oxford (1980).

8. King, G. M., In *Nitrogen Cycling in Coastal in Coastal Marine Environments* (*SCOPE 33*). Blackburn, T. H. and Sorenson, J. (Eds). John Wiley and Sons, Chichester (1988).

9. Wheeler, P. A. and Hellebust, J. A., *Plant Physiology*, **67** (1981), 367.

10. Yancey, P. C., Clark, M. E., Hand, S. C., Bowlus, R. D. and Somero, G. N., *Science*, **217** (1982), 1214.

11. Quinn, P. K., Charlson, R. J. and Bates, T. S., *Nature*, **335** (1988), 120.

12. Ayers, G. P., and Gras, J. L., *Journal of Geophysical Research*, **88**, 10 (1983), 655.

13. Liss, P. S. and Galloway, J. N., In *Interactions of the Biogeochemical Cycles of C, N, P and S'*. Wollast, R. (Ed). Springer-Verlag, Berlin (1992).

14. kulley, F. E., Yang, J. P., Mazina, K. and Daniel, F. B., *Environmental Science and Technology*, **18**, 10 (1984), 787.

15. Mirvish, S. S., *Toxicology and Applied Pharmacology*, **31** (1975), 325.

16. Garside, C., Hill, G. and Murray, S., *Limnology and Oceanography*, **23** (1978), 1073.

17. Hara, H., Motoike, A. and Okazaki, S., *Analyst*, **113** (1988), 113.

18. Searle, P. L., *Analyst*, **109** (1984), 549.

19. Harbin, A.-M. and van den Berg, C. M. G., *Analytical Chemistry*, **65**, 23 (1993) 3411.

20. Jones, R. D., *Limnology and Oceanography*, **36**, 4 (1991) 814.

21. Gibb, S. W., PhD Thesis, University of East Anglia (1994).

22. Tavakkol, A. and Drucker, D. B., *Journal of Chromatography* (*Biomedical Applications*), **274** (1983), 37.

23. Lundstrom, R. C. and Racicot, L. D., *Journal of the Association of Official Analytical Chemists*, **66**, 5 (1983), 1158.

24. Yang, X.-H., Lee, C. and Scranton, M. I., *Analytical Chemistry*, **65** (1993), 572.

25. Di Corcia, A., Samperi, R. and Severini, C., *Journal of Chromatography*, **170** (1979), 325.

26. Delene, M., Mathiasson, L. and Jonsson, J. A., *Journal of Chromatography*, **207** (1981), 37.

27. Kuwata, K., Yamazaki, Y. and Uebori, M., *Analytical Chemistry*, **52**(1980).

28. Bag, S. P., *Talanta*, **32**, 8b (1985), 779.

29. McAleese, D. L., *Analytical Chemistry*, **59** (1987), 541.

30. Gibb, S. W., Mantoura, R. F. C. and Liss, P. S., *Analytical Chimica Acta* (in press).

31. Quinn, P. K. and Bates, T. S., *Environmental Science and Technology*, **23** (1989), 736.

32. Abdul-Rashid, M. K., Riley, J. P., Fitzsimmons, M. F. and Wolff, G. A., *Analytica Chimica Acta*, **252** (1991), 223.

33. Lee, C. and Olsen, B. L., *Organic Geochemistry*, **6** (1984), 259.

